# Measuring bone stiffness using spherical indentation

**DOI:** 10.1371/journal.pone.0200475

**Published:** 2018-07-12

**Authors:** Oliver R. Boughton, Shaocheng Ma, Sarah Zhao, Matthew Arnold, Angus Lewis, Ulrich Hansen, Justin P. Cobb, Finn Giuliani, Richard L. Abel

**Affiliations:** 1 The MSk Lab, Imperial College London, Charing Cross Hospital, London, United Kingdom; 2 The Biomechanics Group, Department of Mechanical Engineering, Imperial College London, South Kensington Campus, London, United Kingdom; 3 Orthopaedic Surgery Department, Charing Cross Hospital, Imperial College Healthcare NHS Trust, London, United Kingdom; 4 Centre for Advanced Structural Ceramics, Department of Mechanical Engineering and Materials, Imperial College London, South Kensington Campus, London, United Kingdom; Indiana University Purdue University at Indianapolis, UNITED STATES

## Abstract

**Objectives:**

Bone material properties are a major determinant of bone health in older age, both in terms of fracture risk and implant fixation, in orthopaedics and dentistry. Bone is an anisotropic and hierarchical material so its measured material properties depend upon the scale of metric used. The scale used should reflect the clinical problem, whether it is fracture risk, a whole bone problem, or implant stability, at the millimetre-scale. Indentation, an engineering technique involving pressing a hard-tipped material into another material with a known force, may be able to assess bone stiffness at the millimetre-scale (the apparent elastic modulus). We aimed to investigate whether spherical-tip indentation could reliably measure the apparent elastic modulus of human cortical bone.

**Materials and methods:**

Cortical bone samples were retrieved from the femoral necks of nineteen patients undergoing total hip replacement surgery (10 females, 9 males, mean age: 69 years). The samples underwent indentation using a 1.5 mm diameter, ruby, spherical indenter tip, with sixty indentations per patient sample, across six locations on the bone surfaces, with ten repeated indentations at each of the six locations. The samples then underwent mechanical compression testing. The repeatability of indentation measurements of elastic modulus was assessed using the co-efficient of repeatability and the correlation between the bone elastic modulus measured by indentation and compression testing was analysed by least-squares regression.

**Results:**

In total, 1140 indentations in total were performed. Indentation was found to be repeatable for indentations performed at the same locations on the bone samples with a mean co-efficient of repeatability of 0.4 GigaPascals (GPa), confidence interval (C.I): 0.33–0.42 GPa. There was variation in the indentation modulus results between different locations on the bone samples (mean co-efficient of repeatability: 3.1 GPa, C.I: 2.2–3.90 GPa). No clear correlation was observed between indentation and compression values of bone elastic modulus (*r* = 0.33, *p* = 0.17). The mean apparent elastic modulus obtained by spherical indentation was 9.9 GPa, the standard deviation for each indent cycle was 0.11 GPa, and the standard deviation between locations on the same sample was 1.01 GPa. The mean compression apparent elastic modulus was 4.42 GPa, standard deviation 1.02 GPa.

**Discussion:**

Spherical-tip indentation was found to be a repeatable test for measuring the elastic modulus of human cortical bone, demonstrated by a low co-efficient of repeatability in this study. It could not, however, reliably predict cortical bone elastic modulus determined by platens compression testing in this study. This may be due to indentation only probing mechanical properties at the micro-scale while platens compression testing assesses millimetre length-scale properties. Improvements to the testing technique, including the use of a larger diameter spherical indenter tip, may improve the measurement of bone stiffness at the millimetre scale and should be investigated further.

## Introduction

Bone stiffness is of crucial importance in orthopaedic surgery and dentistry, both at the time of insertion of a cementless, press-fit implant [1,2] in ensuring a stable press-fit without fracturing the bone and in the long-term, as a difference in the stiffness of the bone and the implant stiffness can lead to the bone around the implant becoming thinner and more porous, a process called stress shielding [3]. This thinner and more porous bone can be more prone to fracture and fractures of the bone around orthopaedic implants occur in over 7% of cementless hip replacement patients in the long term [4]. By better assessing bone stiffness surgical complications in the short and long-term may be reduced [5]. Currently, though, there is no reliable method of measuring the stiffness of a patient’s bone *in vivo* [6]. In this study, spherical indentation is investigated as a potential tool for measuring cortical bone stiffness.

**Fig 1:**
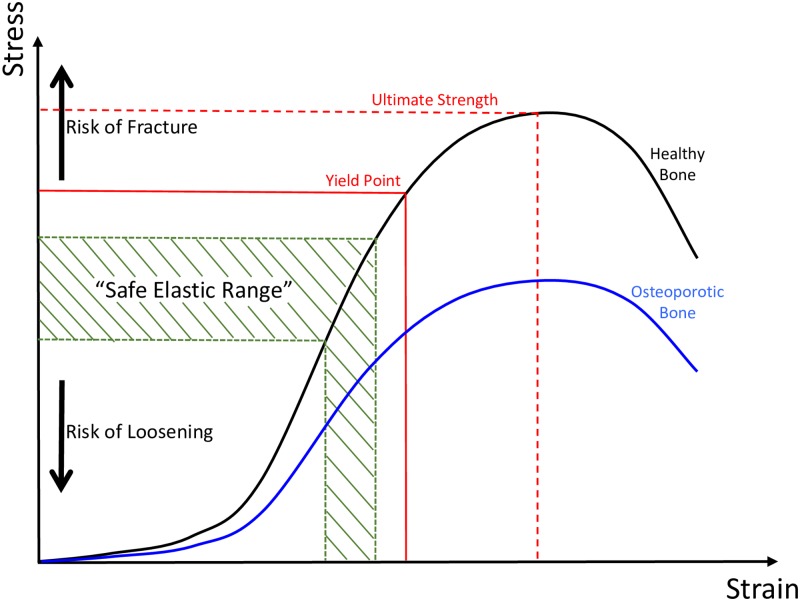
Stress-strain curve. Stress-strain curve demonstrating the proposed “safe elastic range” of bone deformation during press-fit cementless joint replacement surgery. A “safe elastic range” is demonstrated. A comparison is shown between healthy bone (black curve) and more porous, osteoporotic bone (blue curve).

The stiffness of a structure is its ability to resist deformation and is dependent on the material stiffness, known as the elastic modulus, as well the geometry of the structure [7]. The elastic modulus of a material is calculated from the steepness of the linear elastic portion of the stress-strain curve. When bone is stressed beyond this elastic range and past the yield point (Fig 1), it begins to undergo plastic (permanent) deformation. If the stress continues to be applied the ultimate strength (Fig 1) of the bone will be reached and fracture will begin to occur beyond this point [7].

In joint replacement surgery, the press-fit stability of a cementless implant can only be reliably assessed with knowledge of the stiffness of the individual patient’s bone (including time-dependent properties), the implant stiffness and the difference in size and shape between the implant and the prepared bone [1,2]. For a press-fit implant to be stable the patient’s bone needs to be stressed within its elastic range so that there is elastic deformation of the bone. This elastic deformation leads to the bone more firmly gripping the implant due to increased radial stresses applied to the implant by the bone, leading to a reduced chance of either acetabular cup loosening or femoral stem subsidence in total hip arthroplasty that would require revision surgery [2]. We propose that there is a safe window of elastic deformation of the bone, or safe elastic range (Fig 1): If too little stress is applied the bone will not undergo enough elastic deformation, leading to a reduction in the radial stresses applied to the implant by the bone, known as the “elastic grip” [1], potentially leading to implant loosening and early failure [1,8], which can lead to the need for revision surgery. If too much stress is applied the bone will undergo deformation beyond the elastic region on the stress-strain curve, resulting in the bone fracturing [9], either at the time of surgery or early in the post-operative period [2]. To be able to predict this safe elastic range the elastic modulus (the material-level stiffness), volume and shape of the patient’s bone must be known [1,2,10].

Bone stiffness is also very important over the longer term, both in orthopaedics and dental surgery [11]. Bone needs to be loaded to stay strong and if the bone around an orthopaedic implant is shielded from load, the bone will begin to remodel, becoming thinner and more porous. This process is known as stress shielding and occurs most commonly around stiff femoral stem implants in hip replacement devices over the long term [3,12–14]. By closer matching the stiffness of implants to bone stiffness, stress shielding may be reduced [13].

Currently, there is no available reliable method of calculating the stiffness of a patient’s bone *in vivo* [6]. Bone is anisotropic [15], meaning its mechanical properties vary depending on the mechanical direction is tested in, and hierarchical [16], therefore its material properties depend upon the length scale it is being tested at [17]. The scale used should reflect the clinical problem, whether it is fracture risk, a whole bone problem, or implant stability, at the millimetre-scale. Indentation, an engineering technique involving pressing a hard tipped material into another material with a known force [5,18], may be able to assess the apparent elastic modulus of bone (bone stiffness at the millimetre scale) [6,19] but, as of yet, there has been no study that has assessed if the bone elastic modulus measured by indentation at this scale compares favourably to other methods of measuring the elastic modulus at this length scale [5]. Nanoindentation, when combined with bone structural information from high-resolution computed tomography (CT) images, has been shown to accurately predict millimetre-scale mechanical properties of cortical bone [20]. This study used Synchrotron CT to determine the bone structural information [20], though, so this technique cannot be adapted for measuring the elastic modulus bone *in vivo*. Reference point indentation (RPI) is an indentation technique that was developed for *in vivo* testing of bone mechanical properties [21]. RPI has been designed to principally measure the indentation distance increase (IDI) between successive indentations in the same place, a potential surrogate for bone toughness [19]. A handheld RPI instrument has been developed, the Osteoprobe, but this does not currently provide any measure of the elastic modulus of bone [19] as it currently only measures the “Bone Material Strength Index” [22,23]. Therefore, depth-sensing, spherical, microindentation was assessed in this study. Spherical indentation has the advantage over sharp-tip indentation that at small loads and displacements deformation is almost entirely elastic, with plastic deformation occurring at high loads and displacements [24–27].

In a systematic review on microindentation in 2017, there were no studies found that compared spherical indentation with other mechanical testing techniques for measuring stiffness [5]. The aim of this study, therefore, was to assess whether spherical indentation alone, without bone structural information obtained from high-resolution imaging, could predict human cortical bone stiffness at the millimetre length scale (the apparent elastic modulus) in osteoarthritic bone samples collected during arthroplasty surgery.

## Materials and methods

### Sample preparation

Femoral heads and necks were retrieved from 19 living donors undergoing hip replacement surgery for osteoarthritis with their consent (10 female and 9 male donors with a mean age of 69 and age range of 46–86 years). Ethical approval for this research was received (Imperial Tissue Bank number R13004a, Wales Research Ethics Committee number 12/WA/0196).

**Fig 2:**
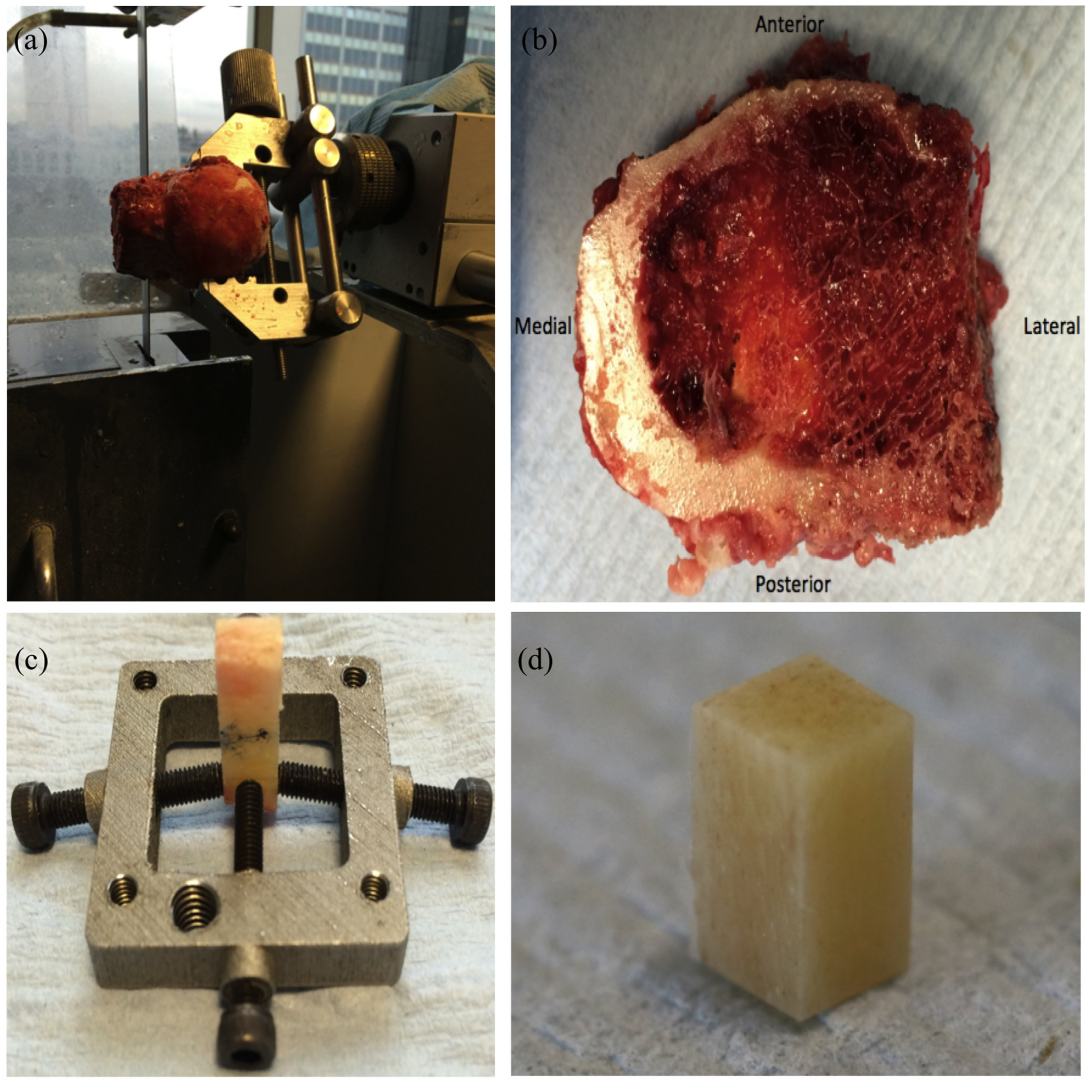
Sample preparation. (**a**) The femoral neck cut from the femoral head using a water-cooled bandsaw. (**b**) Femoral neck after sectioning with the bandsaw, displaying the thicker cortical bone on the medial, calcar area of the femoral neck. (**c**) Custom-made clamp (sample grip) with the four screws gripping the cortical bone section, positioned for the 3 mm-apart parallel cuts. The sample was orientated so that the wafering blade cut the bone perpendicular to the arrow marked on the bone. Arrow corresponds to the direction of the osteons. (**d**) 6x3x3 mm rectangular parallelepiped cortical bone sample from the medial femoral neck cortex. The 6mm length surface is parallel to the direction of the osteons (marked with blue arrow).

The femoral heads and necks were frozen after surgery and thawed prior to testing for one hour in 0.9% saline solution. Freezing of specimens has been shown not to significantly alter the mechanical properties of the tissue [28]. Using a water-cooled bandsaw (Exakt, Scotland) the femoral neck was separated from the femoral head (Fig 2a). Following this, using a high-precision, low-speed saw with a diamond wafering blade (Isomet, Buehler, Germany), a 3x3x6 mm rectangular parallelepiped cortical bone specimen was cut from the medial calcar region of each femoral neck (Fig 2b), so as to have a 2 to 1 length-to-width ratio, as advised by previous research studies [29–31]. To assure parallel surfaces a custom-made additively manufactured steel clamp was designed to hold the specimens whilst cutting (Fig 2c). Dimensions were measured with digital callipers to a precision of ±0.05 mm. The specimens were orientated in the direction of the osteons (Fig 2d).

### Indentation and platens compression testing

**Fig 3:**
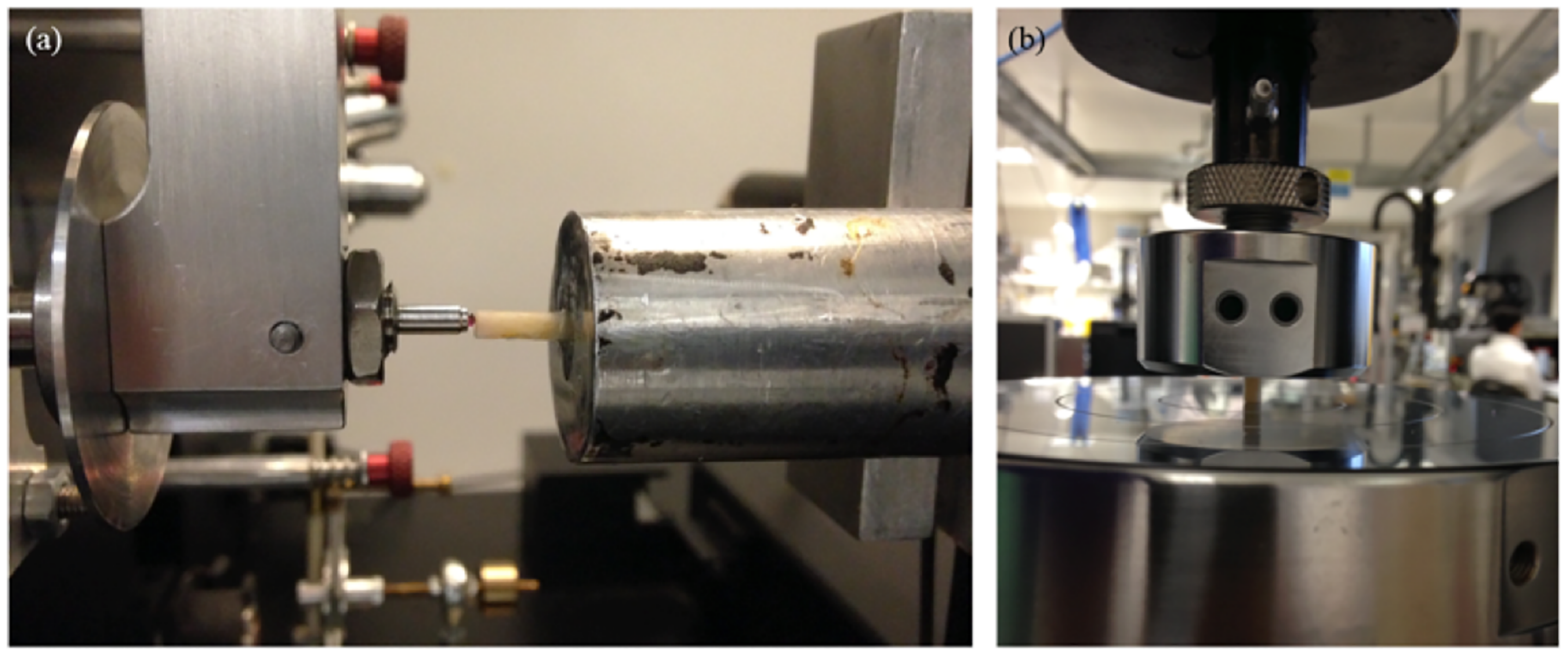
Experimental setup. (**a**) Cortical bone sample mounted in the Micro Materials NanoTest indentation machine. A 1.5 mm diameter, spherical, ruby tip is being used to indent the bone in the direction of the osteons. (**b**) 6x3x3 mm rectangular parallelepiped sample being compressed between two stainless steel platens on a screw-driven Instron materials testing machine.

Specimens underwent indentation testing using a NanoTest 3 indentation machine (Micro Materials Ltd, Wrexham, UK) and the apparent elastic modulus of each specimen was calculated from the unloading curve after indentation using the Oliver-Pharr method [24,32]. A trapezoidal loading pattern was chosen and a spherical, 1.5 mm diameter, ruby indenter tip (Fig 3a) was used to ensure predominantly elastic deformation and to minimise plastic deformation [24–27,33] in the specimen. A zero-load calibration was carried out before each test. Six indentation locations were used on each specimen and 10 indentations were carried out at each location so that 60 indentations were performed on every patient’s bone sample. The spacing between indents was 200 μm on a pre-defined indentation grid.

**Fig 4:**
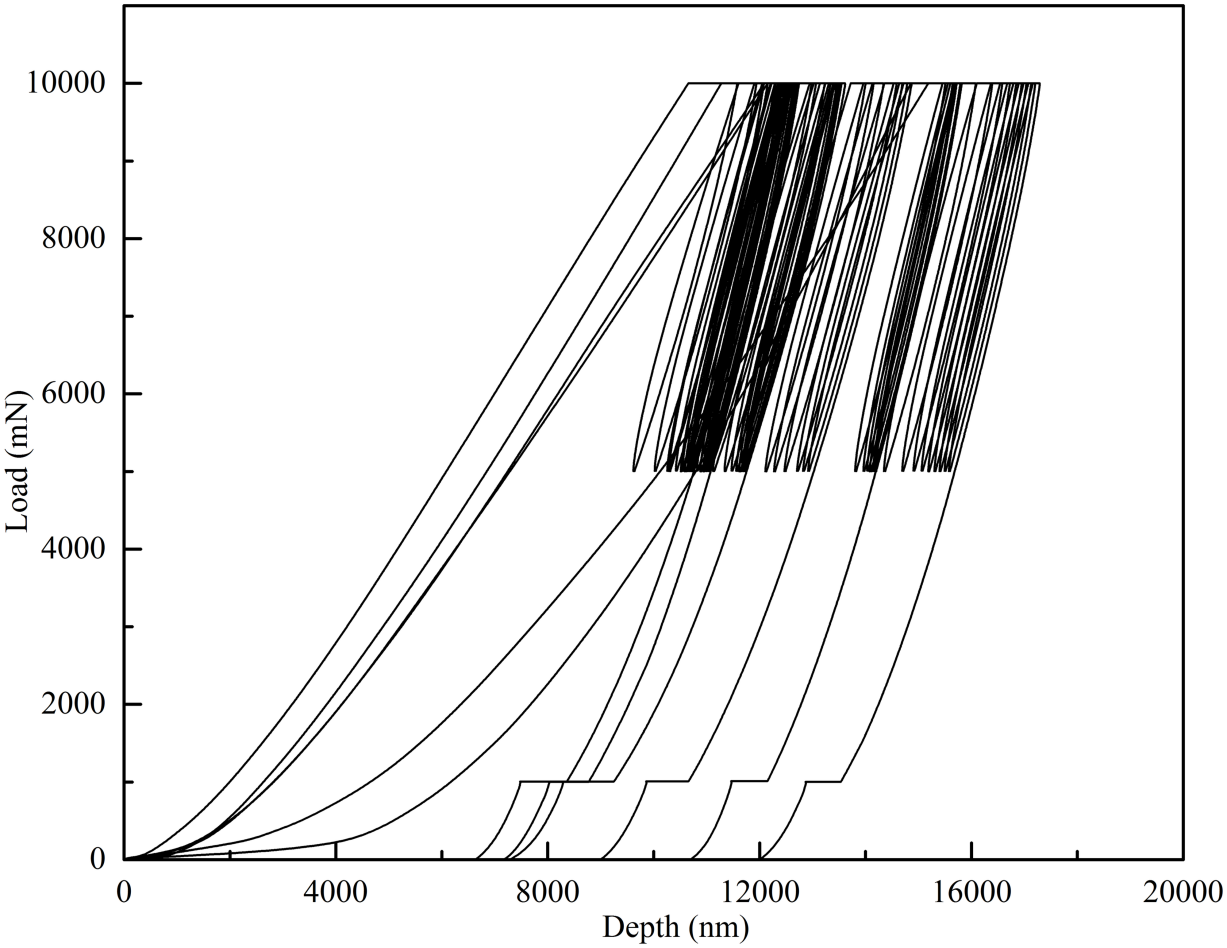
Load-displacement graph of indentation. Load-displacement graph showing indentation in six different locations on the same sample. The bone was loaded to 10000 mN (10 N), there was a 60 second hold period and then it was unloaded to 5 N, followed by immediate reloading to 10 N, with another 60 second holding period at 10 N. There were 10 cycles of loading and unloading at each indent location, followed by complete unloading. The indenter then moved to a new location on a pre-defined grid and there were a further 10 cycles. With 10 cycles of indentation at 6 different locations, 60 indentations were performed in total for each sample.

Load was applied along the longitudinal axis of the bone. A fixed loading rate of 0.1 Newtons per second (N/s) and fast unloading rate of 0.3 N/s were used to minimise the effects of viscoelasticity [34], with a maximum load of 10 N. This load was chosen based on preliminary studies (with the same indenter tip and machines settings) where a 10N load resulted in less than 50 micrometres of displacement, minimising the risk of plastic deformation. At the peak load of every cycle, a dwell period of 60 seconds was used to attempt to exhaust some of the viscoelastic behaviour of the bone. This was a followed by partial (50%) unloading to 5 N, then reloading to 10 N to repeat the process. 10 cycles of loading and partial unloading were performed at each indentation location (Fig 4). Environmental temperature was monitored and kept constant at 24.9°C.

The elastic (Young’s) modulus of bone at the apparent level (millimetre scale level) represents a combination of the intrinsic elastic properties of the bone material as well as the porosity and anisotropy of the total structure [20]. This will be referred to as the apparent modulus or apparent elastic modulus throughout this paper. The apparent elastic modulus of the bone was calculated from the load-displacement data obtained during indentation using the Oliver-Pharr method [24,32]. All data was corrected for machine compliance using data from a tungsten sample and Berkovich tip calibration test. The Micro Materials software, inbuilt into the depth-sensing NanoTest 3 indentation machine (Micro Materials, Wrexham, UK), was used to analyse the data using the Oliver-Pharr method [24,32]. Bone was assigned a Poisson’s ratio of 0.33 [35] and ruby was assigned an elastic modulus and Poisson’s ratio of 420 GigaPascals (GPa) and 0.24, respectively [36].

Full details of the Oliver-Pharr method are in the original paper by, 1992 [32] and the modification to the method for spherical indentation is in the 2004 paper by Oliver and Pharr [24]. Briefly, load (*P*) and displacement (*h*) are automatically recorded by the indentation machine. Three values are taken from the load-displacement graph: maximum load (*P*_*max*_), (maximum displacement) (*h*_max_) and the unloading stiffness, *S*, which is the slope of the upper portion of the unloading curve, d*P*/d*h*. To calculate *S* a power law fitting curve is fitted to the upper portion of the unloading slope of the load-displacement graph. The formula for this is:

$$P=\alpha {\left(h-h_{f}\right)}^{m}$$

Where α and *m* are power law fitting constants. After this, the contact depth, *h*_*c*_, is calculated. For spherical indentation, the contact depth equation is:

$$h_{c}=\frac{h_{\text{max}}+h_{f}}{2}$$

Where *h*_*f*_ is determined from the power law fit from the unloading slope. The indentation area, *A*, is then calculated by:

$$A=2\pi Rh_{c}$$

Where *R* is the radius of the indenter tip, which was 0.75mm in this study. The reduced modulus, *E*_*r*_ is next calculated:

$$E_{r}=\frac{\sqrt{\pi }}{2}\times \frac{S}{\sqrt{A}}$$

The elastic modulus, *E*, is then calculated from the following formula, where *v* is the Poisson’s ratio, and *i* denotes the indenter tip material [24,32]:

$$\frac{1}{E_{r}}=\frac{\left(1-v^{2}\right)}{E}+\frac{\left(1-v_{i}^{2}\right)}{E_{i}}$$

**Fig 5:**
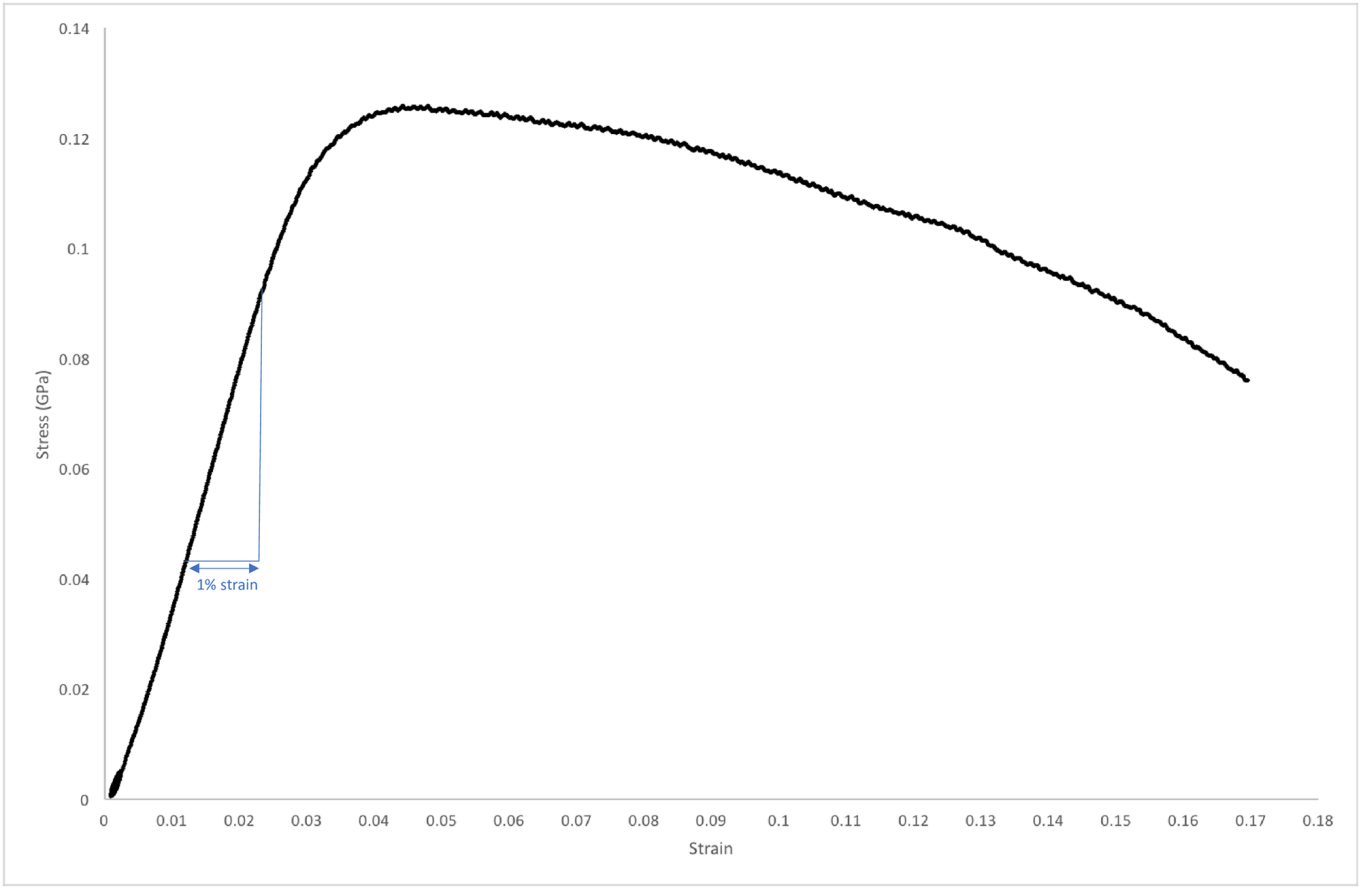
Compression testing stress-strain graph. Graph displaying strain on the x-axis and stress (in GigaPascals) on the y-axis from one of the cortical bone samples that underwent compression testing in the materials testing machine in this study. The elastic modulus was calculated from the slope of a best-fit line at the steepest part of the linear section of the stress-strain curve, over a 1% strain range, which is displayed on the graph.

Following indentation testing, a mechanical testing device (Instron, UK) was used to compress all of the specimens (bulk tissue compression testing). Platens compression testing was performed on a screw-driven Instron materials testing machine (Instron 5565, Instron Ltd, High Wycombe, UK). The samples were placed unconstrained between polished plano-parallel stainless-steel platens and the compressive load was applied in the longitudinal axis of the specimen (Fig 3b). Samples were kept moist during testing. A 5kN load-cell measured load whilst displacement was measured via the machine crosshead displacement. Machine compliance was corrected for using the Bluehill compliance correction software (Instron, USA). Destructive testing was then carried out at a displacement rate of 1.8 mm/minute. This corresponds to a strain rate of 0.005/s, which is considered to be the quasi-static strain rate for bone and the strain rate is recommended to be in this range from previous studies [29,31,37]. The load-displacement data was converted to stress-strain curves using the geometry of the specimens. The apparent elastic modulus was obtained from the slope of the linear elastic portion of the stress-strain curves at the maximum slope of the stress-strain curve by plotting a best-fit straight line over 1% strain, with a varying origin, similar to the method by Keaveny et al., 1997 [31], and Reed and Brown, 2001 [38] (Fig 5).

The data were analysed to assess the repeatability (precision) [39] of indentation for measuring bone elastic modulus and the relationship between the elastic modulus calculated by compression testing when compared to indentation. The repeatability was assessed to estimate indentation measurement error [40] by calculating the coefficient of repeatability [41]. This is the standard deviation of the measurement, multiplied by 1.96 and the square root of 2 [41,42]. It is expressed in GigaPascals (GPa), the unit of original measurement. The coefficient of repeatability was calculated for both repeated indentations in the same locations on the bone samples and for indentations at different locations on the bone sample (for each bone sample there were sixty indentations performed, with ten indentations performed at six different locations). The mean coefficients of repeatability, with confidence intervals, were calculated for indentations in the same locations and different locations using one-sample T-Tests with IBM SPSS Statistics v24 (IBM, USA). The relationship between the apparent elastic modulus calculated by compression testing when compared to indentation was assessed using ordinary least-squares regression to calculate the Pearson’s correlation coefficient, r, using IBM SPSS Statistics v24 (IBM, USA).

## Results

**Fig 6:**
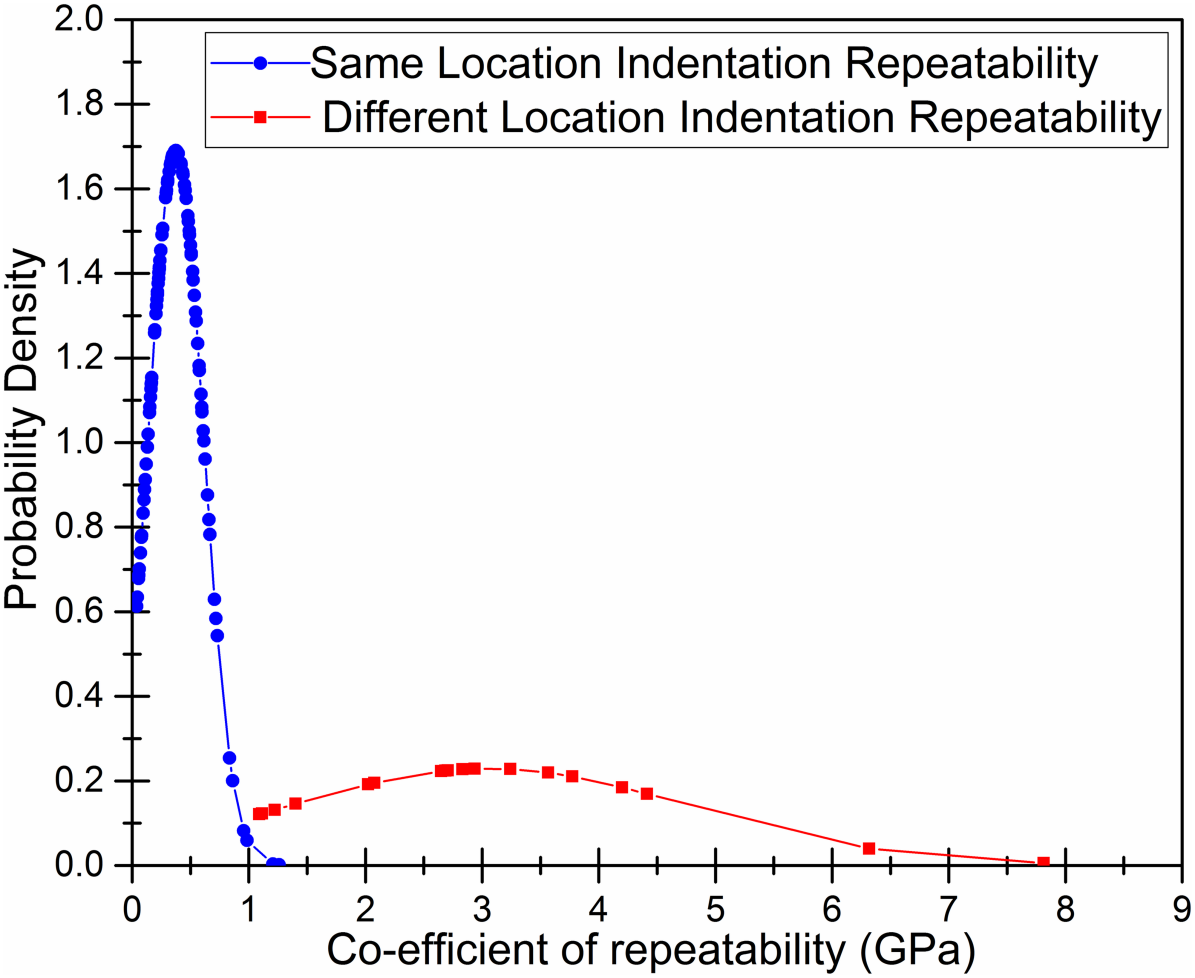
Repeatability of indentation measurements of bone elastic modulus. Distribution plot of the co-efficient of repeatability measurements. The co-efficient of repeatability values (GigaPascals) are on the x-axis. The y-axis displays the relative probability. Two graphs are plotted: In blue, the co-efficient of repeatability values for repeated indentations at the same locations are plotted. There were six different locations on each of the 19 patient bone samples so there are 114 data points for repeated, same location, indentation repeatability co-efficient values. In red, the co-efficient of repeatability values for indentations at different locations are plotted. There were 19 data points, corresponding to the 19 patient samples. The mean co-efficient of repeatability was 0.4 GigaPascals (GPa) (confidence interval (C.I): 0.33–0.42 GPa) for indentations at the same bone locations and 3.1 GPa (C.I: 2.2–3.90 GPa), at different locations on the bone samples.

1140 indents in total were performed on 19 samples from 19 patients. Table 1 displays the sample demographics and a summary of the results. Indentation was found to be repeatable for indentations performed at the same locations on the bone samples with a mean co-efficient of repeatability of 0.4 GigaPascals (GPa), confidence interval (C.I): 0.33–0.42 GPa. There was variation in the indentation apparent elastic modulus results between different locations on the bone samples (mean co-efficient of repeatability: 3.1 GPa, C.I: 2.2–3.90 GPa). The co-efficient of repeatability values for indentations in the same locations and at different locations across the bone samples are displayed graphically in Fig 6.

**Table 1:** Indentation and compression apparent elastic moduli (GPa).

Sample Number	Gender	Age (years)	Indentation Modulus (GPa)	S.D. of Sample (GPa)	Mean S.D. within same Indent Location (GPa)	Compression Modulus (GPa)
**1**	F	46	7.6	0.4	0.1	4.4
**2**	F	51	12.6	1.3	0.1	4.2
**3**	F	62	5.8	0.4	0.1	4.7
**4**	F	67	9.7	0.4	0.1	3.5
**5**	F	73	6.6	1.1	0.1	5.6
**6**	F	78	13.8	0.5	0.1	6.9
**7**	F	79	8.6	1.5	0.1	4.8
**8**	F	80	6.0	1.2	0.1	1.7
**9**	F	83	9.1	0.9	0.2	4.2
**10**	F	85	13.7	0.7	0.1	4.1
**11**	M	56	12.4	0.7	0.1	4.1
**12**	M	57	9.3	0.4	0.1	4.3
**13**	M	58	10.4	1.0	0.2	3.3
**14**	M	59	13.5	0.9	0.1	5.0
**15**	M	65	12.6	2.6	0.2	4.6
**16**	M	76	6.0	1.0	0.1	4.2
**17**	M	76	13.0	2.1	0.1	4.9
**18**	M	77	7.5	0.9	0.1	4.3
**19**	M	81	9.4	1.4	0.1	5.2

The standard deviations (GPa) of the indentation moduli across different locations on the same sample (standard deviation of sample) and the means of the standard deviations of the indentation moduli for each of the six indent locations for each sample are shown. Hence, the first standard deviation represents variation across the bone whereas the second standard deviation represents variation in modulus within the same indent location. The age and sex (male (M) and female (F)) of the patients from which the samples were obtained are displayed.

**Fig 7:**
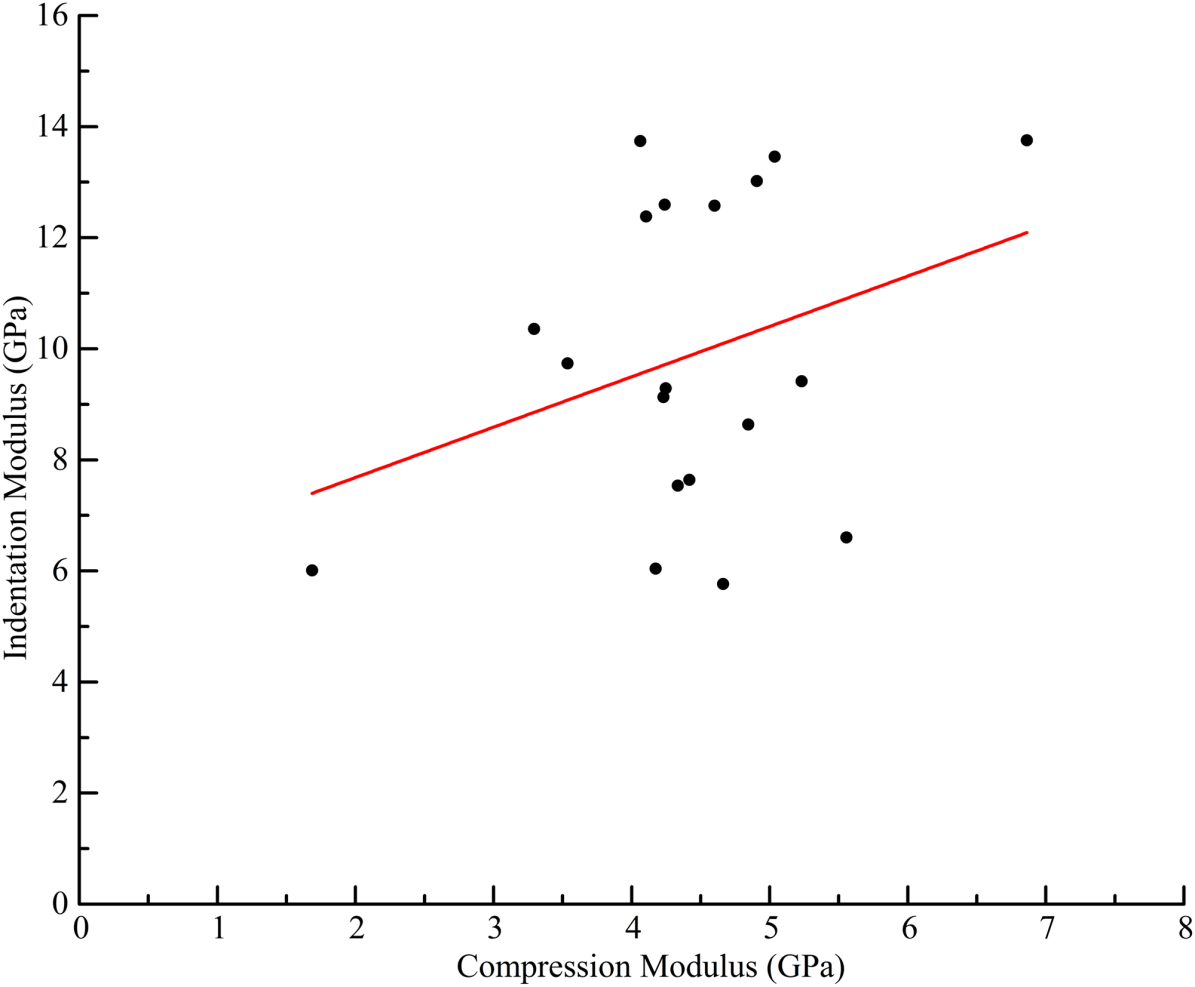
Indentation modulus vs compression modulus scatter plot. Scatter plot of the compression apparent elastic modulus (GPa) compared to the indentation apparent modulus (GPa). The regression line plotted has an *r* value of 0.33, *p* = 0.17.

The mean apparent elastic modulus obtained by spherical indentation was 9.9 GPa, the standard deviation (SD) for each indent cycle was 0.11 GPa, and the standard deviation between locations on the same sample was 1.01 GPa. The mean compression apparent elastic modulus was 4.42 GPa, standard deviation 1.02 GPa. There was no demonstrable correlation (Fig 7) between indentation and compression values of apparent modulus (*r* = 0.33, n = 19, *p* = 0.17).

## Discussion

This study directly compared indentation and platens compression testing for measuring the apparent elastic modulus of 19 cortical bone samples. Spherical-tip indentation was found to be a repeatable test for measuring the apparent elastic modulus of cortical bone, demonstrated by a low co-efficient of repeatability for repeated indentations on the same bone locations in this study. This was the first study showing repeatable results for measuring human cortical bone elastic modulus using a 1.5 mm diameter, spherical indenter tip. This study found that there was quite a marked variation in the apparent elastic modulus measured by indentation at different locations within the same bone samples. This could reflect the heterogeneity of bone [16]. The indentations in different parts of the bone surface may have sampled some of the local porosity and the different relative elastic modulus values of interstitial and osteonal bone [43].

We hypothesised that performing indentation using a 1.5 mm diameter, spherical indenter tip would sample some of the porosity and hierarchical structure of the bone, and the mechanical data obtained from the indentation would correlate with the mechanical properties of bone at the apparent (millimetre or mesoscale) level. Indentation could not, however, reliably predict cortical bone apparent elastic modulus determined by platens compression testing in this study. We propose that there were two main reasons for the variation in indentation results at different bone locations and for the observed lack of correlation between indentation and compression testing results.

**Fig 8:**
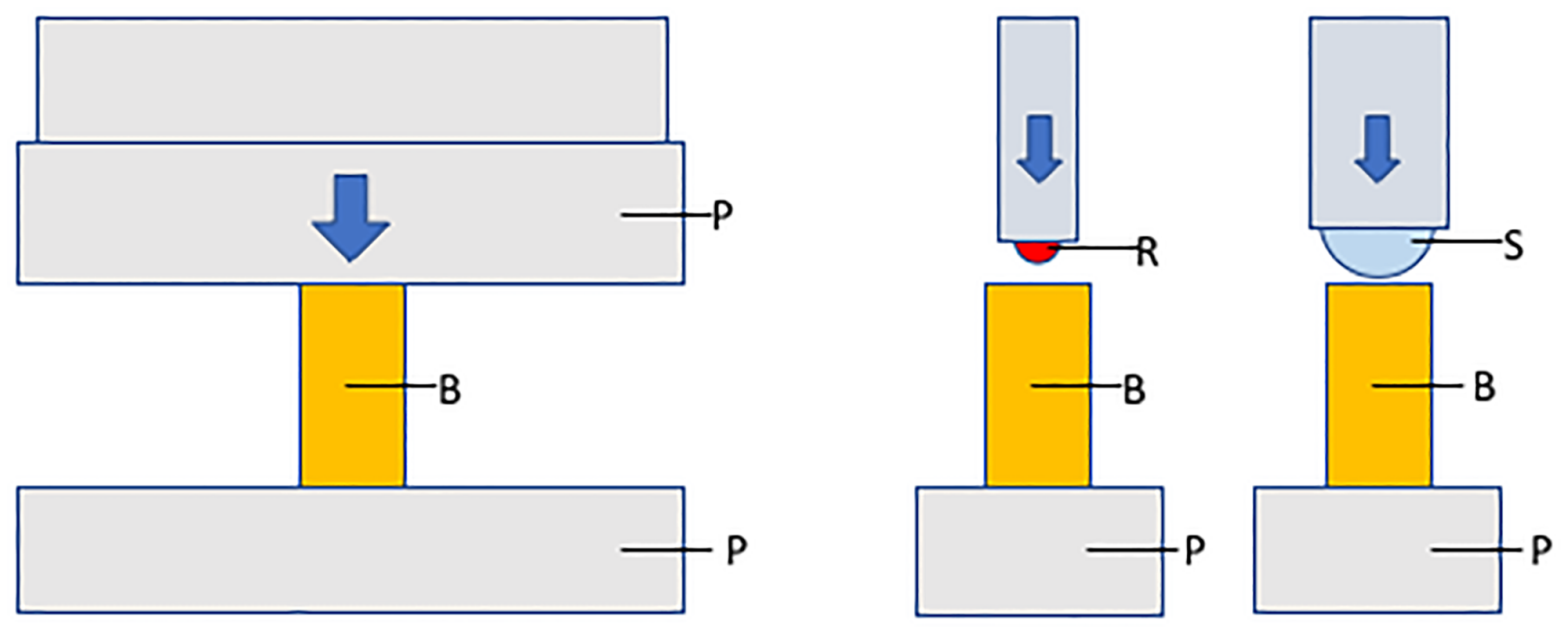
Schematic of compression testing, compared to indentation testing. Left: Bone (B) undergoing compression testing between two stainless steel platens (P). The blue arrow indicates the load being applied. Centre: Bone undergoing indentation using the 1.5 mm diameter, ruby, spherical indenter tip (R) used in this study. Right: Bone undergoing indentation using a proposed larger diameter, spherical indenter tip (S).

Firstly, spherical indentation and platens compression testing are testing cortical bone at two different length scales. A 1.5 mm diameter, spherical indenter tip, with a calculated bone contact zone diameter of approximately 200 microns using the Oliver-Pharr analysis technique [24,32], may not have been sufficiently large to sample enough of the hierarchical properties [16,44,45] of cortical bone. Local variation in cortical porosity [33] may have led to some of the observed scatter in results. Cortical porosity has been shown to be the major determinant of mesoscale (millimetre scale) elastic properties [46,47] and not accounting adequately for porosity by indentation may have led to the lack of correlation in our results. Porosity may also account for the variation in elastic modulus values across the bone surfaces because some indentations may have occurred within porous regions, and others in denser, stiffer areas of the same bone sample. Additionally, porosity may have contributed partially to the lower modulus values obtained from compression testing compared to indentation testing as the apparent modulus of a structure will be reduced substantially with increasing porosity [48], whereas the indentation technique may only have been sampling a small area of the bone between pores. When performing indentation on cortical bone, if a larger indenter tip is used more of the cortical bone pores will be included in each indentation. Hence, if a larger tip is used more of the bone structure will be represented, which may closer resemble what is being tested when the whole bone sample is compressed in platens compression testing. This is illustrated in Fig 8.

Secondly, testing errors may have occurred during indentation and compression testing. Sample dehydration has been shown to increase the stiffness of bone [49] and there may have been some sample drying out towards the end of indentation testing, potentially leading to higher apparent modulus values from indentation when compared to compression testing. In a nanoindentation study Hengsberger et al. found indentation moduli ranged from 7.4 to 18.5 GPa in hydrated bone, whilst in dry bone the indentation moduli ranged from 11.1 to 31.6 GPa [49]. We tried to reduce sample dehydration by storing the samples in 0.9% saline-soaked tissue paper between testing but did not use a liquid cell for indentation as the indentation machine we used performs indentations horizontally, rather than vertically, making liquid testing very challenging.

Elastic modulus values from compression testing in this study were lower than most values reported for human cortical bone in the general literature [50]. We propose that the main reason for the lower modulus values was because our cortical bone samples were taken from the femoral neck whereas most cortical bone measurements reported in the literature are from samples of bone from the mid-shaft of the femur [50]. The elastic properties of cortical bone have a large reported range throughout the body [50–53] and the elastic modulus of human cortical bone has been reported to range from 7.4 to 31.6 GPa, due to variation in individual bones tested as well as testing conditions and techniques [50]. Indeed, proximal femoral cortical bone specimens have been shown to be 23% less stiff than diaphyseal (mid-shaft) femoral cortical bone specimens [53]. A similar reduced stiffness in the proximal femoral cortical bone, relative to the femoral diaphyseal bone, was reported in a nanoindentation study [51].

Another potential explanation is that the mean age of the patients was 69 and cortical bone porosity is known to increase with age [54], leading to an associated decrease in the stiffness of bone at the millimetre scale with age [46]. Bone material itself may become stiffer with age [55] but, due to increasing porosity with age, stiffness at the millimetre scale gradually decreases [46]. In a Synchrotron computed tomography study of femoral neck bone from elderly female patients the cortical porosity was found to range from 5.0% to 38.9% (mean 15.9%) [56]. In addition, platens compression testing may have underestimated bone elastic modulus in our study, as demonstrated in other studies [31]. We used the inbuilt compliance correction software on the Instron testing rig but this may not have sufficiently reduced the underestimation.

Indentation techniques have been shown to be very useful for measuring the elastic properties of bone at the tissue level [50]. In a study in which nanoindentation elastic modulus data were combined with high-resolution Synchrotron computed tomography (CT) calculations of bone porosity the prediction of the elastic modulus of cortical bone at the millimetre-scale (apparent level) was very good [20]. Synchrotron CT imaging is not feasible *in vivo*, though, due to the extremely high radiation dose [57]. Nanoindentation-derived mechanical properties do not always correlate well with mechanical properties of the whole bone [58] and this is likely due to it only sampling the tissue-level mechanical properties in a small area, without taking into account changes in porosity or other features in the hierarchical structure of bone [58].

Granke et al., 2014, explored using reference point indentation (RPI) to measure bone mechanical properties. Using the benchtop BioDent system from Active Life Scientific, they measured the unloading slope of cortical bone from the femoral diaphysis and performed three-point bending tests to calculate the apparent-level mechanical properties. No correlation between mechanical properties measured by RPI and the apparent modulus measured by three-point bending [19] was found. This agrees with the findings from our study, although their study differs in the indentation system used and the use of a sharp-tip rather than spherical tip indenter. Dell’Ara et al. compared the benchtop BioDent RPI system with depth sensing microindentation for measuring the material properties of bone. The unloading slope measurements from RPI correlated with the indentation moduli from depth sensing microindentation but the indentation distance increase (IDI) measurements did not correlate with any depth sensing microindentation measurements [59]. The handheld RPI device, the Osteoprobe from Active Life Scientific, only currently measures the “Bone Material Strength Index” and does not record the unloading slope [23], in contrast to the BioDent. The handheld device, therefore, does not record a measurement of bone elastic modulus currently.

Mirzaali et al., 2016, also used sharp-tip indentation to test femoral diaphyseal cortical bone specimens, but with a Berkovich indenter tip in an Ultra Nano Hardness Tester, rather than RPI. The moduli measured by indentation did not correlate with the moduli obtained from tensile testing of the bone specimens. Like in our study, Mirzaali et al. explained that the lack of correlation between indentation and whole specimen tensile and compression testing material properties was likely due to porosity of the bone samples [60].

Spherical-tip indentation is an attractive potential technique for measuring the bone elastic modulus due to the reduced plastic deformation of the bone compared to sharp-tip indentation [61]. In our study, it was possible that some plastic deformation did occur in the bone samples as a result of the relatively high displacements. With spherical indentation, Oliver and Pharr predict first yielding (plastic deformation) to occur when the indentation depth (*h*) divided by the indenter tip radius (*R*) exceeds 1.4 x 10^−4^ [24]. The maximum indentation depth in this study was 50 micrometres, with an indenter tip radius of 750 micrometres, leading to the *h*/*R* ratios in our samples being greater than the ratio predicted by Oliver and Pharr. Therefore, it is likely that some plastic deformation occurred in the samples.

Spherical tip indentation has been used to measure the mechanical properties of cortical bone in the patella but this study did not have another mechanical testing technique to compare indentation testing against [62]. In a study by Oyen et al., spherical indentation of bone with varying indenter tip sizes was investigated [33]. This study did not compare indentation to other methods of mechanical testing, though. To improve on our study using spherical tip indentation, adaptations to the indentation technique including the use of a larger spherical tip that will reflect more of the local porosity of the bone and may lead to measurements that better represent how bone behaves in compression at the millimetre length-scale.

Alongside indentation, other techniques are being developed which may potentially soon become safe and reliable methods of measuring cortical bone elasticity *in vivo*. High-resolution computed tomography (CT) techniques are improving rapidly and micro-CT techniques are currently the acknowledged standard for measuring bone structure *ex vivo* [18]. The radiation dose is currently a concern for *in vivo* use of high-resolution CT but as techniques improve CT may soon become a reliable and safe method of measuring *in vivo* cortical bone porosity [54,63], a reasonable surrogate marker for bone elasticity [46]. Ultrasound-based techniques are also becoming promising potential methods of measuring cortical bone elasticity and porosity *in vivo*[64–66].

In conclusion, spherical-tip indentation was found to be a repeatable test for measuring the apparent elastic modulus of human cortical bone, demonstrated by a low co-efficient of repeatability in this study. We were unable to demonstrate a correlation between indentation measurements of cortical bone elastic modulus and cortical bone elastic modulus measured by platens compression testing. Improvements to the testing technique, including the use of a larger diameter spherical indenter tip, may improve the measurement of bone’s elastic modulus at the millimetre scale and should be investigated further.

## Supporting information

S1 Table Indentation testing raw data and calculated compression testing apparent elastic moduli. (XLSX)

S2 Table Compression testing raw data. (XLSX)

S3 Table Raw data from the compression testing of bone sample 1 in the materials testing machine. The raw data and calculations of the elastic modulus are shown in the table and two graphs.
(XLSX)
